# Analysis of glycoalkaloid distribution in the tissues of mealworm larvae (*Tenebrio molitor*)

**DOI:** 10.1038/s41598-024-67258-5

**Published:** 2024-07-17

**Authors:** Magdalena Joanna Winkiel, Szymon Chowański, Maria Sulli, Gianfranco Diretto, Małgorzata Słocińska

**Affiliations:** 1 Department of Animal Physiology and Developmental Biology, Faculty of Biology, Institute of Experimental Biology, Adam Mickiewicz University in Poznań, Uniwersytetu Poznańskiego 6, 61-614 Poznan, Poland; 2 Italian National Agency for New Technologies, Energy and Sustainable Development ENEA, Via Anguillarese 301, 00123 Roma, Italy

**Keywords:** Solanine, Chaconine, Detoxification, Plant secondary metabolites, Insect, Mass spectrometry, Analytical chemistry, Chemical biology, Chemical biology, Physiology

## Abstract

Solanine (SOL) and chaconine (CHA) are glycoalkaloids (GAs) produced mainly by *Solanum* plants. These plant secondary metabolites affect insect metabolism; thus, they have the potential to be applied as natural plant protection products. However, it is not known which GA concentration induces physiological changes in animals. Therefore, the aim of this study was to perform a quantitative analysis of SOL and CHA in the larvae of *Tenebrio molitor* using LC‒MS to assess how quickly they are eliminated or metabolised. In this experiment, the beetles were injected with 2 μL of 10^−5^ M SOL or CHA solution, which corresponds to a dosage range of 0.12–0.14 ng/mg body mass. Then, 0.5, 1.5, 8, and 24 h after GA application, the haemolymph (H), gut (G), and the remainder of the larval body (FB) were isolated. GAs were detected in all samples tested for 24 h, with the highest percentage of the amount applied in the FB, while the highest concentration was measured in the H sample. The SOL and CHA concentrations decreased in the haemolymph over time, while they did not change in other tissues. CHA had the highest elimination rate immediately after injection, while SOL slightly later. None of the GA hydrolysis products were detected in the tested samples. One possible mechanism of the detoxification of GAs may be oxidation and/or sequestration. They may be excreted by Malpighian tubules, with faeces or with cuticles during moulting. The results presented are significant because they facilitate the interpretation of studies related to the effects of toxic substances on insect metabolism.

## Introduction

Plants produce many compounds, called primary metabolites, which are essential for their growth, development, and metabolism. During primary metabolic reactions, byproducts called plant secondary metabolites (PSMs) are created. PSMs are frequently involved in defence mechanisms activated in response to many different stressors, such as changes in environmental conditions, infections, or herbivore feeding^1^. Alkaloids are one of the major groups of PSMs, with approximately 10,000 reported derivatives^2^. In particular, steroidal glycoalkaloids (GAs), such as solanine (SOL) and chaconine (CHA), contain glycoside residues attached to nitrogenous aglycone and are produced mainly by *Solanaceae* crop plants, such as potato (*Solanum tuberosum* L.) and tomato (*Solanum lycopersicum* L.). GAs can be found in almost all plant organs, including leaves, stems, roots, and tubers^3^. GAs demonstrate high biological activity^4^; they are toxic to cells from various groups of organisms; thus, GAs are a defence against herbivores and pathogens. On the other hand, the cytotoxic, antioxidant, antiviral, and antimicrobial properties of GAs can be used in the drug industry^5,6^.

GAs are biologically active substances, and their effects on cells have been reviewed by many researchers^7,8^. For example, GAs are strong inhibitors of acetylcholinesterase and butyrylcholinesterase, which catalyse the hydrolysis of the neurotransmitter acetylcholine in the nervous system^8^. They can affect the cell division process by inducing the ornithine decarboxylase enzyme and can modulate Ca^2+^ and Na^+^ transport across cell membranes. Moreover, GAs form complexes with cholesterol, which leads to cell disruption and leakage of cell contents. Some reports indicate teratogenic effects in animals caused by GAs, with CHA being more toxic than SOL^7^. The toxic effect of GAs depends on many factors, such as the type of carbohydrate chain in the structure, the presence of a nitrogen atom in the GA ring, and the pH value^7^. In mammals, the LD_50_ values for GAs are similar in different species. In rodents, the metabolism of GAs is defined by low absorption, rapid excretion, and hydrolysis to alkaloids, which are less toxic. Due to poor absorption, the intraperitoneal LD_50_ values are much lower than the values calculated after GAs are consumed orally. For example, the intraperitoneal LD_50_ for SOL is 34 mg/kg body weight in mice, while for orally consumed GAs, the LD_50_ is more than 1000 mg/kg body weight^7^.

GAs also have a wide range of insecticidal activities^4,9,10^. The addition of pure GAs and leaf extracts of *S. tuberosum* and *S. lycopersicum* to the culture media caused malformations and reproductive disturbances in *Drosophila melanogaster* M.^11^. Specifically, SOL, CHA, and tomatine were found to be toxic to the red rust flour beetle (*Tribolium castaneum* H. ) and rice weevil (*Sitophilus oryzae* L.)^12^. Similarly, SOL and its extract from tomato leaves administered with food affected the fertility, fecundity, and survival of *Galleria mellonella* L.^13,14^. The impact of solasonine and *S. nigrum* L. extracts on this species was also studied. These substances were found to affect the composition of haemolymph metabolites as well as the ultrastructure of the fat body and midgut cells^15^. GAs and their extracts also exhibit cardioactive properties that were shown in *T. molitor* and *Zophobas atratus* B. beetles^16,17^. Sublethal effects of GAs, such as disturbed development, food intake, and reproduction, were observed in *T. molitor* after the addition of pure GAs and *S. nigrum* fruit extract to food^18^. The injection of GAs and tomato leaf extract into *T. molitor* larvae affects lipid metabolism^19^; in turn, the administration of *S. nigrum* fruit extract modulates insect immune system activity^20^ in this species.

As GAs affect insect metabolism, they have the potential to decrease insect survival or, by disturbing development and fecundity, reduce insect populations; therefore, they can be applied as natural plant protection products^22^. However, it is not known exactly which GA concentrations induce the abovementioned physiological changes in animals because, in most studies, GAs were administered in food in the extract form or/and the incubation time was arbitrarily chosen. Therefore, the aim of this study was to perform a quantitative analysis of these compounds at specific time points after the injection of GAs into the bodies of the larvae of *T. molitor* to assess how quickly they are eliminated or metabolised by the beetle. Additionally, the survival of larvae after GA injection was measured to assess the potential lethality and long-term effects of GA treatment. *T. molitor* is a popular model organism for biochemical, physiological, and environmental studies. The genome of this species was already published, its breeding is easy, and the physiology is well known, which facilitates the interpretation of its results. Moreover, *T. molitor* is a grain storage pest.

Additionally, SOL and CHA are the main GAs of potato tubers and are among the most important agricultural products. According to FAO data, 376 million tons of potatoes were produced worldwide in 2021 (FAOSTATs, 2021). The Colorado potato beetle (*Leptinotarsa decemlineata* S.), potato ladybird (*Henosepilachna vigintioctopunctata*, F.), and potato tuber moth (*Phthorimaea operculella*, Z.) are among the most dangerous potato pests that cause significant food loss^21^. As some species seem to be resistant to GAs, it is crucial to discover the mechanisms of GA action in insects because they might be useful for developing new strategies against crop pests.

## Results

**Figure 1:**
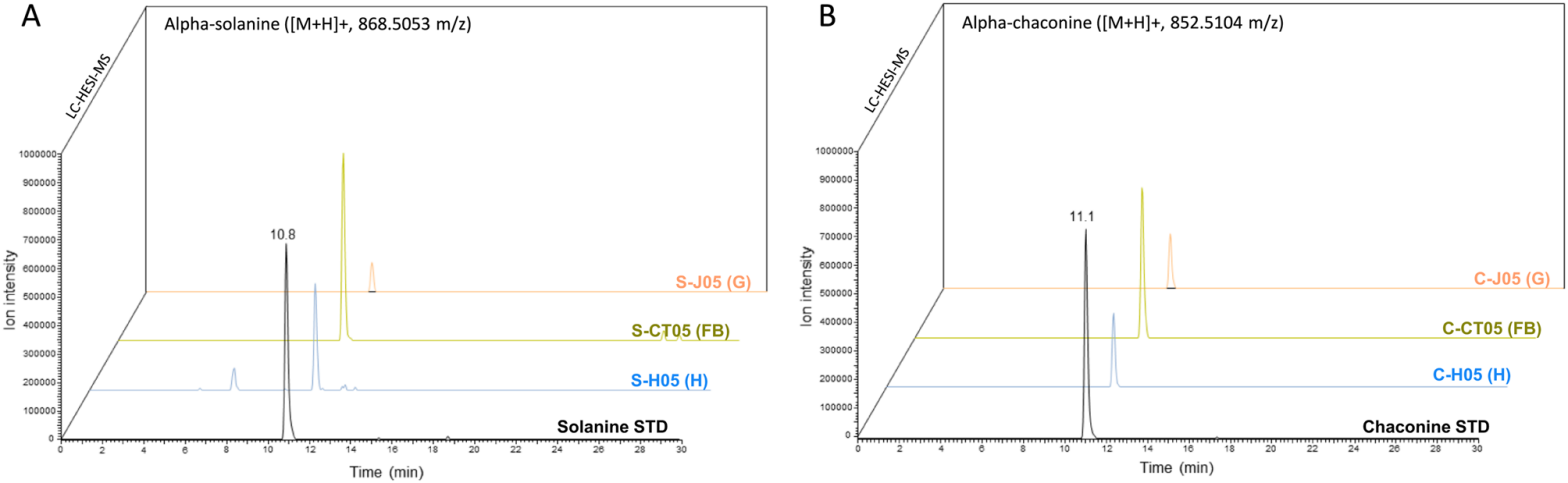
Accurate MS spectra of SOL (**A**) and CHA (**B**) extracted from the haemolymph (H), gut (G) and remaining part of the larvae (FB) 0.5 h after injection and analysed by LC-HESI_MS alongside authentic standards (STD).

The SOL and CHA amounts in the haemolymph (H), gut (G), and remaining part of the larvae (FB) from the insects injected with the tested GAs were measured using LC-HRMS. The sample mass spectrum data are shown in Fig. 1. For SOL and CHA, the m/z values of the M + H ions were 868.5053 and 852.5104 (Dppm < 3), respectively. The calibration curves were established with appropriate analytical standards. The retention time for SOL was 10.8 min, while it was 11.1 min for CHA.

**Figure 2:**
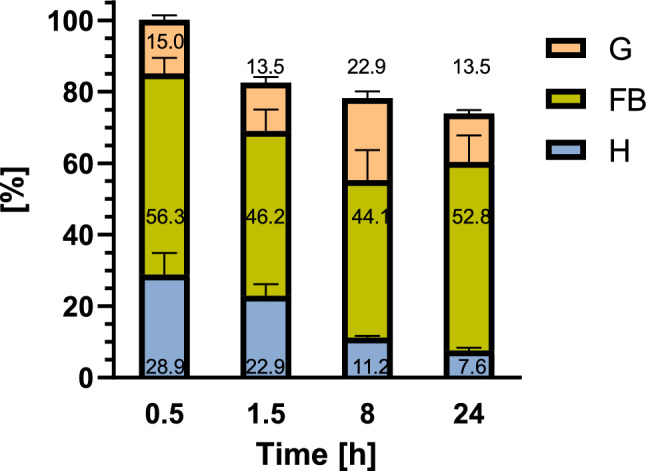
Percentage of total applied SOL in each of the samples obtained from the haemolymph (H), gut (G) and remaining part of the larvae (FB) 0.5, 1.5, 8 and 24 h after injection. The data are shown as the mean and SEM. The pooled samples were used with *n* = 4, and for each experimental variant, three independent replicates were performed.

At 0.5 h after SOL application, most of the applied GA (69.5 ± 2.95 ng) (~ 100%) remained in the insect body (Fig. 2). During the next hour, the total amount of SOL started to decrease by approximately 12.2 ± 2.50 ng (~ 17.5%), and the greatest decrease in total SOL content occurred over time. Therefore, we observed a delay in the elimination of SOL from the insect body. During the next 6.5 h, we observed a further decrease averaging only 3.0 ± 4.00 ng (~ 4.4%) and another 3.0 ± 2.19 ng (~ 4.3%) in the quantity of SOL at 8 h of analysis. After 24 h, 51.3 ± 8.76 ng of SOL (~ 73.9% of the administered amount) remained in the insect body.

The distribution of SOL in different tissues also changed over time. At the first time point (0.5 h after injection), the lowest amount of total GA was located in the gut (10.4 ± 1.55 ng, ~ 15.0% of the applied amount of GA). Twofold more SOL was detected in the haemolymph (20.0 ± 7.24 ng, ~ 28.9% of the applied SOL), and 39.1 ± 5.23 ng (~ 56.3% of the injected GA amount) was spread across the remaining parts of the larval body, mainly in the fat body. One hour later, the SOL level in the gut decreased to 9.4 ± 1.91 ng (which constitutes ~ 13.5% of the applied amount) but increased to 15.9 ± 2.30 ng (~ 22.9%) at the next time point and finally reached 9.4 ± 1.17 ng (~ 13.5%) after 24 h. In the samples obtained from the haemolymph, the amount of SOL systematically decreased to 15.9 ± 3.97 ng (~ 22.9%), 7.7 ± 0.67 ng (~ 11.1%), and 5.3 ± 0.90 ng (~ 7.6%) 1.5, 8, and 24 h after application, respectively. In the FB sample, we observed the lowest fluctuation in SOL content. At each time point, the SOL content in the samples was similar and reached values of 32.1 ± 7.26 ng, 30.6 ± 10.15 ng, and 36.7 ± 8.89 ng (between ~ 44.1 and ~ 52.8% of the applied SOL) after 1.5, 8, and 24 h of injection, respectively.

**Figure 3:**
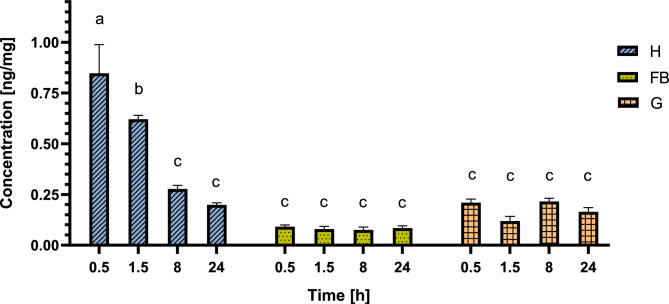
Concentration of SOL in the analysed tissues as ng/mg in the haemolymph (H), gut (G) and remaining part of the larvae (FB) 0.5, 1.5, 8 and 24 h after injection. The data are shown as the mean and SEM. The pooled samples were used with *n* = 4, and for each experimental variant, three independent replicates were analysed via two-way ANOVA.

The greatest total amount of SOL at the 0.5 time point was detected in the FB when the total tissue mass was taken into account (Fig. 2). Knowing the total mass of each tissue used for sample preparation, we also calculated the concentration of SOL in each tissue. The results (Fig. 3) showed that in FBs, the concentration was low and constant (between 0.08 ± 0.018 and 0.09 ± 0.015 ng/mg). This indicates a low affinity of SOL for that tissue. The highest concentration 0.5 h after GA injection was observed in the haemolymph (0.85 ± 0.244 ng/mg). This concentration was more than 9 times greater than that in FB (0.09 ± 0.015 ng/mg) and four times greater than that in the gut (0.21 ± 0.031 ng/mg) at that time point. At 24 h, the concentration of SOL in the haemolymph decreased by more than 4 times (to 0.20 ± 0.018 ng/mg), but at each time point, it was still greater than that in the FB (Fig. 3). The SOL concentration in the gut ranged between 0.16 ± 0.035 and 0.22 ± 0.028 ng/mg and did not change with time. The ratio of the SOL concentration in different tissues changed from 1.0:9.3:2.3 (FB:H:G) to 1.0:7.8:1.5, 1.0:3.7:2.8, and 1.0:2.4:2.0 (Table 1) at each subsequent time point. The equalization of the concentrations of SOL in the haemolymph (H) and gut (G) samples at the 8 h and 24 h time points may be the reason why the elimination of SOL slowed significantly after 8 and 24 h, whereas the lack of significant changes in the SOL concentration in the FB may have resulted from the fact that during the entire tested period, the SOL concentration in that tissue was lower than that in the haemolymph.

**Table 1:** SOL concentration ratio in the haemolymph (H) and gut (G) to the concentration in the remaining part of the larvae (FB) at 0.5, 1.5, 8 and 24 h after GA injection.

Time after GA injection [h]	Sample		
	FB	H	G
0.5	1.0	9.3	2.3
1.5	1.0	7.8	1.5
8	1.0	3.7	2.8
24	1.0	2.4	2.0

For clarity, the concentration of FB was considered to be 1.

**Figure 4:**
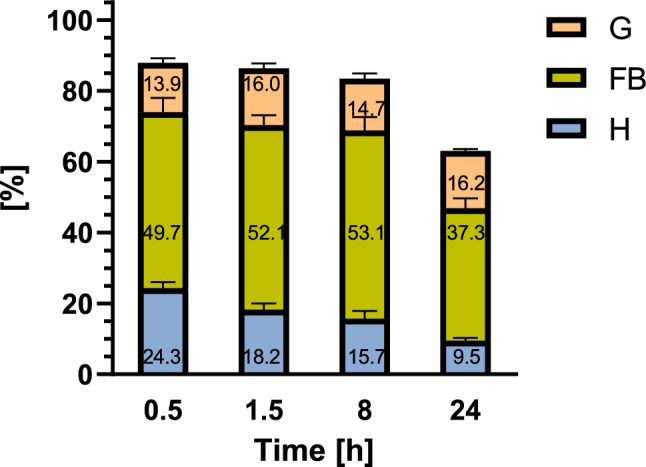
Percentage of total applied CHA in each sample obtained from the haemolymph (H), gut (G) and remaining part of the larvae (FB) 0.5, 1.5, 8 and 24 h after injection. The data are shown as the mean and SEM. The pooled samples were used with *n* = 4, and for each experimental variant, three independent replicates were performed.

At the first time point (0.5 h after injection), 59.9 ± 1.75 ng (~ 87.9%) of CHA was applied to the entire insect body (68.2 ng); therefore, the elimination of CHA started just after application (Fig. 4). During the next hour, the CHA concentration decreased to 58.8 ± 4.34 ng (~ 86.3%), and at the next time points, we measured 56.9 ± 9.45 ng (~ 83.0%) and 43.0 ± 4.16 ng (~ 63.1%) of applied CHA as still present in the insect body 8 and 24 h after application, respectively.

Regarding the distribution of GAs in the tested tissues, at the beginning of the experiment (0.5 h variant), the lowest total level of GA was in the gut (9.5 ± 1.50 ng, ~ 13.9% of the applied amount). A nearly twofold greater level of CHA was detected in the haemolymph (16.6 ± 2.04 ng, ~ 24.3% of applied CHA), and 33.9 ± 4.72 ng (~ 49.7% of GA amount) was detected in the FB sample. In the gut, the CHA concentration increased slightly 1.5 h after injection to 10.9 ± 1.64 ng (~ 16.0% of the applied amount) but decreased to 10.0 ± 1.77 ng (~ 14.7%) at the next time point and reached 11.0 ± 0.62 ng (~ 16.2%) again after 24 h. In the haemolymph samples, the CHA concentration decreased systematically during the whole experiment to 12.4 ± 2.06 ng (~ 18.2%), 10.7 ± 2.61 ng (~ 15.7%), and 6.5 ± 0.91 ng (~ 9.5%) 1.5, 8, and 24 h after injection, respectively. In the FB samples, the amount of CHA increased at 8 h after application, reaching 36.2 ± 3.63 ng (~ 53.1%), while it decreased at 24 h to 25.5 ± 3.28 ng (~ 37.3% of the amount of injected CHA).

**Figure 5:**
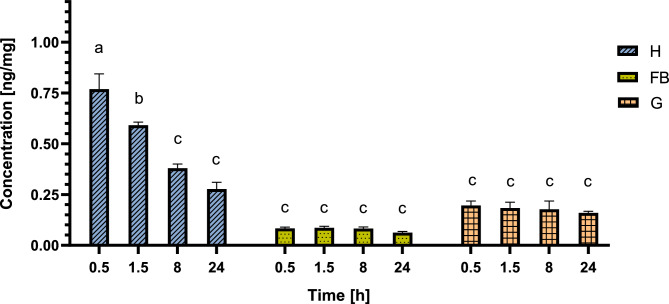
Concentration of CHA in the analysed tissues as ng/mg in the haemolymph (H), gut (G) and remaining part of the larvae (FB) 0.5, 1.5, 8 and 24 h after injection. The data are shown as the mean and SEM. The pooled samples were used with *n* = 4, and for each experimental variant, three independent replicates were analysed via two-way ANOVA.

Similar to SOL, when we considered the whole tissue mass, the greatest amount of CHA was detected in the FB samples (Fig. 4). However, the lowest concentration, expressed as ng/mg, was calculated in these samples and was between 0.06 ± 0.010 and 0.09 ± 0.013 ng/mg (Fig. 5). There were no significant changes in the CHA concentration during the whole experiment either in the FB or in the gut samples (0.16 ± 0.014–0.20 ± 0.037 ng/mg). The highest GA concentration at all time points tested was observed in the haemolymph (0.28 ± 0.058–0.77 ± 0.129 ng/mg). This value was more than 9 times greater than that in FB (0.08 ± 0.012 ng/mg) and almost 4 times greater than that in the gut (0.20 ± 0.037 ng/mg) at the 0.5 time point (Table 2). The greatest changes in CHA concentration over 24 h were also detected in that tissue (almost 3 times). The proportion of GA concentration in gut samples (G) with other tissues increased at the 24-h time point. The ratio of CHA concentration in different tissues changed from 1.0:9.3:2.4 (FB:H:G) to 1.0:6.8:2.1, 1.0:4.6:2.1, and 1.0:4.4:2.5 (Table 2) at each subsequent time point.

**Table 2:** CHA concentration ratio in the haemolymph (H) and gut (G) to the concentration in the remaining part of the larvae (FB) at 0.5, 1.5, 8 and 24 h after GA injection.

Time after GA injection [h]	Sample		
	FB	H	G
0.5	1.0	9.3	2.4
1.5	1.0	6.8	2.1
8	1.0	4.6	2.1
24	1.0	4.4	2.5

For clarity, the concentration of FB was considered to be 1.

**Figure 6:**
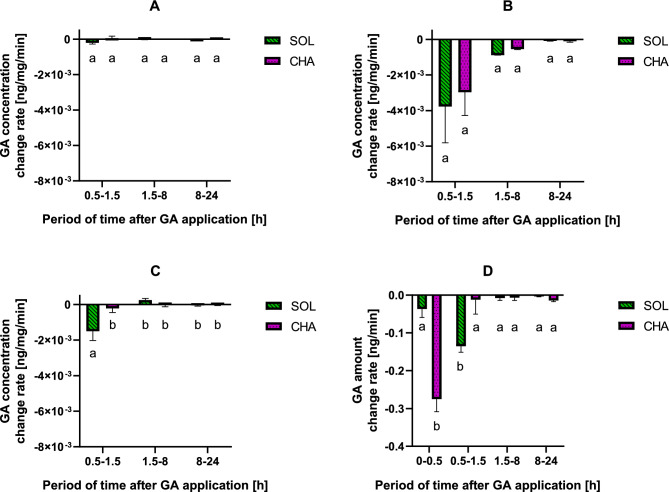
Changes in the SOL and CHA concentrations in each tissue sample, FB (**A**), H (**B**), and G (**C**) at a rate of ng/mg/min and in the whole larva (**D**) at a rate of ng/min 24 h after GA injection. Values above zero indicate accumulation, while negative values indicate the elimination rate compared with the previous time period. The lower the negative values are, the greater the elimination rate. Higher positive values indicate a higher accumulation rate. The pooled samples were used with n = 4, and for each experimental variant, three independent replicates were performed.

The concentrations of SOL and CHA in the samples changed at different rates (Fig. 6). SOL was eliminated from FB at 0.5–1.5 h after injection at a rate of 2.01·10^–4^ ± 1.319·10^–4^ ng/mg/min (Fig. 6A). Furthermore, the elimination rate tended to be greater during this period than between 1.5 and 8 h (9.79·10^–6^ ± 3.233·10^–5^ ng/mg/min). During the last period (8–24 h), GAs slowly accumulated (9.12·10^–6^ ± 7.160·10^–6^ ng/mg/min). On the other hand, CHA accumulated in FBs during all the test periods, at the beginning of the experiment (0.5–1.5 h) at a rate of 6.04·10^–5^ ± 1.941·10^–4^ ng/mg/min, during the next test period at 1.14·10^–6^ ± 1.092·10^–4^ ng/mg/min, and in the last test period at 1.84·10^–5^ ± 3.264·10^–5^ ng/mg/min.

The elimination rate of SOL in the haemolymph (Fig. 6B) tended to be greatest during the first test period of 0.5–1.5 h (3.78·10^–3^ ± 3.532·10^–3^ ng/mg/min), and it was almost 19 times greater than that in FB. In the subsequent timespans, SOL was removed from the haemolymph more slowly (8.82·10^–4^ ± 8.588·10^–6^ and 8.13·10^–5^ ± 3.081·10^–5^ ng/mg/min). The changes in the CHA elimination rate in the haemolymph were very similar to those in the SOL but were slightly lower (2.97·10^–3^ ± 2.278·10^–3^, 5.44·10^–4^ ± 5.035·10^–5^, and 1.07·10^–4^ ± 8.223·10^–5^ ng/mg/min in the subsequent tested periods). However, the changes were not statistically significant.

During 0.5–1.5 h, the elimination rate of SOL in the gut (Fig. 6C) was the greatest (1.50·10^–3^ ± 9.079·10^–4^ ng/mg/min). However, it accumulated between 1.5 and 8 h at a rate of 2.46·10^–4^ ± 1.609·10^–4^ ng/mg/min (*p* < 0.001). In the next time span, SOL was eliminated again at a rate of 5.27·10^–5^ ± 6.186·10^–5^ ng/mg/min. CHA was eliminated from the gut during the whole experiment at the highest rate during the 0.5–1.5 ng/mg/min period (2.18·10^–4^ ± 4.066·10^–4^ ng/mg/min). During the first test period, SOL was eliminated from the gut faster than CHA (*p* < 0.01).

The changes in the calculated and detected amount of GAs in the whole larvae (the sum of GAs in FB, G, and H) are shown in Fig. 6D. Overall, SOL and CHA were eliminated from the larval body throughout the entire experiment. During the first test period, CHA was eliminated faster (0.275 ± 0.0584 ng/min) than during the next period (0.012 ± 0.0659 ng/min) (*p* < 0.0001), and the highest rate was observed during the whole experiment. SOL was eliminated the fastest at 0.5–1.5 h after injection, at a rate of 0.135 ± 0.0278 ng/min. This value is greater than that in the first test period (*p* < 0.05) and greater than that in the next one (*p* < 0.01). CHA was eliminated more than 7 times faster (0.275 ± 0.0584 ng/min) than SOL (0.037 ± 0.0386 ng/min) at 0.5 h after injection (*p* < 0.0001). However, in the next test period (0.5–1.5 h), the elimination rate of SOL was more than 11 times greater (0.135 ± 0.0278 ng/min) than that of CHA (0.012 ± 0.0659 ng/min) (*p* < 0.01). During the subsequent time spans (1.5–8 h and 8–24 h), the elimination rates of GAs were much slower than before and reached values between 0.003 ± 0.0023 and 0.014 ± 0.0157 ng/min.

**Figure 7:**
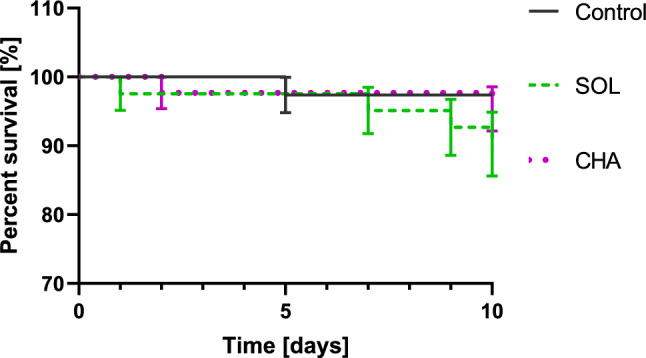
Larval survival after GA injection. The error bars are shown as the means with SEs, n = 15; for each experimental variant, three independent replicates were performed, and the log-rank test was performed (Mantel‒Cox).

The survivability of the *T. molitor* larvae after 2 μL of SOL or 10^−5^ M CHA was analysed for 10 days (Fig. 7). In the three replicates, one dead larva was observed in the control with physiological saline application, four larvae in the variant with SOL and two larvae after CHA injections. However, there were no significant changes in survival compared to the control. The first dead larva in the experimental variant with SOL was observed one day after injection, while for CHA treatment, it was observed after two days.

## Discussion

Xenobiotics are chemical compounds that are not natural components of a living organism but are exposed to them. They undergo metabolic processes, including absorption, distribution, biotransformation, and excretion. Xenobiotics can enter insects through the cuticle or eggshell or through the oral route. Then, the detoxification process begins. The compounds undergo modifications and degradations, including oxidation‒reduction reactions that increase their solubility and facilitate their elimination from the organism. Subsequently, the modified xenobiotic is excreted first from cells and finally from the organism by different types of transporters^23^.

Many insects that are crop pests are exposed to glycoalkaloids. The purpose of this study was to perform a quantitative analysis of GAs at specific time points in different tissues after their injection to assess how they are distributed and accumulated throughout the insect as well as how quickly they are metabolised and eliminated by the insect.

Under natural conditions, GAs can enter the insect body with food. Most studies involve feeding insects plants containing GAs or the preparation of an artificial diet supplemented with GAs^13–15,18,24^. However, without knowledge about the exact concentrations of these compounds in insects, it is impossible to understand the precise mechanism of action of GA. Thus, in this research, we applied SOL and CHA by injection to deliver the exact amount of the compound to the larvae. We tested samples of haemolymph, gut, and samples obtained from the remaining part of the insect body, which mainly consisted of the fat body, Malpighian tubules, and cuticle. Insect haemolymph is composed of fluid plasma containing haemocytes, and it circulates around other tissues in the insect body. The fat body fills the body cavity surrounding the digestive tract. It is immersed in the haemolymph, which facilitates the exchange of metabolites. It is the main organ involved in intermediary metabolism in insects. Therefore, it is not surprising that the applied GAs were detected in the fat body and haemolymph (Figs. 2, 4). However, the results also indicate that the GAs were transported to the insect gut (Figs. 2, 4). They might be transported from the haemolymph directly or/and with Malpighian tubules. This may be one of the explanations for the GA loss in the haemolymph over time. The Malpighian tubules are long tubes that are connected to the gut between the midgut and hindgut. They build up the excretory system, which is responsible for maintaining homeostasis^25^.

When a xenobiotic enters an insect, it may undergo different detoxification reactions. The type of process depends on the chemical nature of the compound. GAs are classified as glycosides because they are composed of carbohydrate chains, and the aglycone is connected by a glycosidic bond. Glycosides, in turn, are acetal compounds with the general formula R_2_C(OR′)_2_^10^. Acetals are obtained during the nucleophilic addition of two alcohol molecules to an aldehyde or ketone in the presence of an acid catalyst^26^. This condensation reaction is called acetalisation. Acetals are stable to bases, reducing agents, and nucleophiles; however, they break down in an acidic environment^26^. SOL and CHA are produced in plants through the cholesterol pathway through the glycosylation of carbohydrates (carbonyl compounds) with solanidine (alcohol)^7,8^. Additionally, GAs are derived from alkaloids.

**Figure 8:**
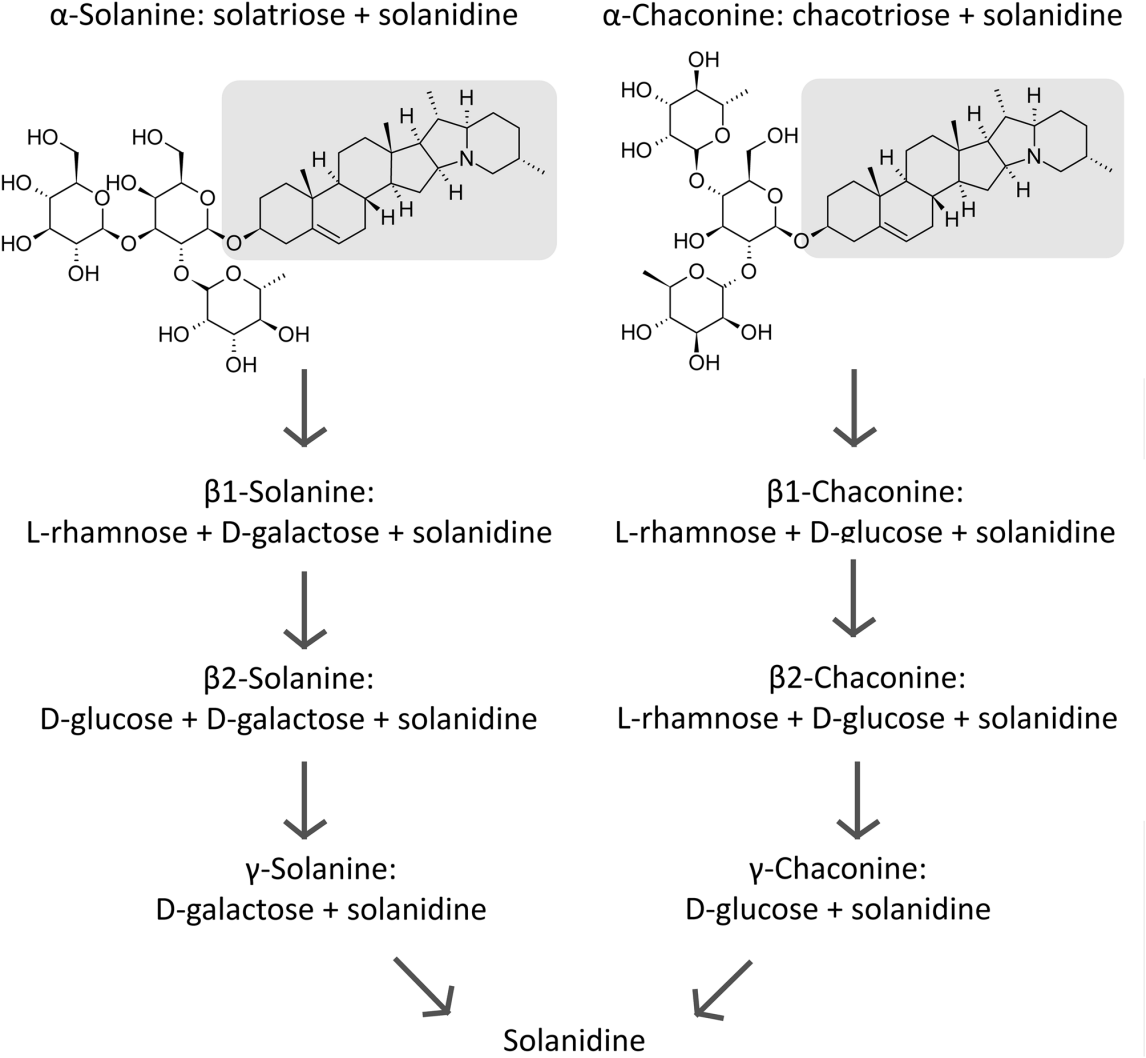
Hydrolysis products of GAs.

The biotransformation of GAs involves the hydrolysis process, which leads to different products. Carbohydrate groups are susceptible to hydrolysis in acids as well as to hydrolysis catalysed by enzymes. First, the detachment of particular sugar molecules leads to the formation of β-compounds, followed by the formation of γ-derivatives. The aglycon part, called solanidine, remains when all sugar chains are removed from the SOL or CHA molecule (Fig. 8)^7,8^. Hydrolysis of the glycosidic bond results in the loss of GA activity^10^; thus, biotransformation is an ability of many organisms (to avoid toxicity) as well as of different plant species (to eliminate autotoxicity risk), although nitrogen-containing chains often show high resistance to transformation. Many bacterial species have the ability to metabolise GAs by detaching carbohydrate groups or oxidising hydroxyl groups^27^. Plants and phytopathogenic fungi contain glycosidases that hydrolyse GA molecules. However, it is not known whether mammalian glycosidases also have such properties^7,8^. Glycosidases have been identified in insects of various orders, such as Orthoptera, Hymenoptera, and Coleoptera^28^. These enzymes were also reported in adults of *T. molitor*^29^ as well as in larvae^30^. However, contrary to expectations, none of the GA hydrolysis products were detected in this study. One possible explanation is that glycosidases present in *T. molitor* larvae have high substrate specificity and do not react with GA compounds.

Insects have developed complex protection systems for defence against different xenobiotics^23^. Some toxic molecules can be metabolised into easily excreted compounds and eliminated from the body by the excretory system. Other xenobiotics can be modified into safer chemicals to facilitate their accumulation in tissues^25^. The tested GAs did not cause lethal toxicity for 10 days after application (Fig. 7); thus, insects can use a variety of strategies to deal with xenobiotics. One of the physiological adaptations of the organism to prevent poisoning is rapid intestinal passage, which protects against the accumulation of toxins^31^. Usually, cytochrome P-450 is involved in detoxification processes. It catalyses the oxidation of different xenobiotics, such as phytochemicals and insecticides^32^. For example, nicotine (another alkaloid of *Solanaceae* plants) given in food to *Manduca sexta* larvae induces cytochrome P-450 in the midgut epithelium^33^. Nicotine present in the haemolymph is metabolised, and the product of its oxidation is actively transported to the Malpighian tubules via a nonspecific alkaloid pump and is subsequently excreted^25,33^. The active transport of alkaloids to urine has also been reported in the larvae of *Rhodnius prolixus* and *Pieris brassicae*^34^. However, G-strophanthin, a cardiac glycoside, is also actively transported in *Zonocerus variegatus*, while in *Locusta migratoria,* it moves passively into the Malpighian tubules^35^. The detoxification enzymes act in the fat body and Malpighian tubules; however, they are the most active in the insect midgut^25,36^. Some species, such as the butterfly *Danaus plexippus,* maintain oxidising conditions in the midgut to defend against plant-derived compounds^37^. In *Spodoptera litura,* many detoxification-related genes were upregulated after tomatine treatment. In addition to the P450 genes, glutathione S-transferases, ABC transport enzymes, UDP-glucosyltransferases, and carboxylesterases were also upregulated, mainly in the midgut and fat body^36^. The molecular mechanisms involved in the action of all these enzymes in the *Spodoptera* genus were described in a review^38^, while the regulation of their expression in insects was described in the study of Amezian et al.^39^. In addition to the oxidation system, xenobiotics that enter the insect body can be sequestered and stored in the cuticle, glands or haemolymph^25,40^. For example, *Oncopeltus fasciatus* (Hemiptera) can sequester g-strophanthin^41^. Thus, one possible mechanism for the detoxification of GAs is oxidation and/or sequestration.

Various xenobiotics are removed from insects in different ways. The elimination path depends on the type of detoxification process that takes time. This is the first study to analyse changes in GA concentration in *T. molitor* tissues over time. The change in the applied SOL percentage at different time points is shown in Fig. 2. The oxidation/excretion processes did not occur during the first 30 min after application because most of the GA was detected in the samples. At the end of the experiment (24 h after injection), 73.9% of the applied SOL remained in the larvae. On the other hand, the CHA content was much lower than the SOL percentage after 0.5 h (87.9% of the applied CHA) and 24 h after injection (63.1%) (Fig. 4). The results indicate that CHA is eliminated immediately after injection, while there is a delay in SOL elimination. Moreover, GA excretion processes are relatively slow because 24 h is not enough to remove all GA from the larval organism.

As expected, the highest percentage of applied GA among the tissues tested was in the FB sample (Figs. 2, 4), which mainly contained a fat body and Malpighian tubules, due to the function of these tissues described above. Furthermore, these results are consistent with other studies because the lipid droplets in the fat body of the *T. molitor* larvae*,* as well as the *G. mellonella* larvae treated with the extract of *S. nigrum,* solasonine, and solamargine showed decreased homogeneity and lysis of the content of lipid droplets^15,18^. Thus, GAs can alter fat body structure. Moreover, SOL, CHA, and tomatine affect lipid metabolism^19^. Despite GA delivery by injection through the cuticle, the compounds were also detected in the gut tissue. This indicates that GAs can be transferred to the gut, where they are involved in GA metabolism and/or elimination. This finding was also reported by Li et al.^24^, who studied GA accumulation in the potato tuber moth *P. operculella*. In this study, the concentrations of GAs applied to food-containing insects were analysed in the head, foregut, midgut, hindgut, cuticula, and faeces of larvae. In the insects fed potato leaves, SOL was detected in the faeces and midgut, while CHA was excreted in the faeces and accumulated in the hindgut, head, midgut, and cuticle (in order of decreasing GA content). In the insects fed 0.3% GAs containing an artificial diet (1 mL of CHA and 0.75 mL of SOL), SOL was found in the midgut and faeces, while CHA was detected in the midgut, hindgut, faeces, head, and cuticle (in order of decreasing GA content). None of these GAs were detected in the foregut. Unfortunately, neither the haemolymph nor the fat body was studied therein. The excretion of GAs in faeces might be the most effective method for their detoxification. These results are consistent with our suggestions that SOL and CHA are excreted by *T. molitor* mainly through the faeces and cuticle.

The concentration of GAs in insects depends on the type of tissue (Figs. 3, 5) and are eliminated at different rates (Fig. 6). The concentration of GAs in the FB sample was relatively low and did not change with time (Figs. 3, 5), thus showing a low affinity for that tissue. Moreover, their concentration change rates were almost constant (Fig. 6A). In the haemolymph, the SOL and CHA concentrations decreased within 8 h after application (Figs. 3, 5), and the elimination rate tended to be the highest at the beginning of the experiment (Fig. 6B). In the gut, similar to that in the FB sample, the GA concentration was also quite low, and there were no changes in GA concentration with time (Figs. 3, 5). On the other hand, during the first tested period (0.5–1.5 h), SOL was eliminated at the fastest rate in the whole experiment and significantly faster than CHA (Fig. 6C). A possible explanation for this finding might be that GAs present in the haemolymph are transported to the gut (directly or/and with the Malpighian tubules), maintaining a constant, maximum level. This result can also be explained by the direct transfer of these compounds to the cuticle. Considering the whole insect, the tested GAs were eliminated from the larval body throughout the entire experiment (Fig. 6D). Thus, in addition to GA transport between tested tissues, SOL and CHA must be eliminated outside the body, for example, via faeces. These results corroborate the findings of^24^, who reported GA excretion in faeces as well as during moulting. It is possible that the amount of GA in the gut as well as in the fat body would decrease when it reached the saturated concentration in the haemolymph. The observed changes might also be attributed to the sequestration of some of these plant secondary metabolites in the insect body.

In the present study, SOL and CHA, which are toxic to the larvae of *T. molitor,* were injected into beetles, and the percentage amounts of GAs were analysed in different tissues within 24 h at specific time points. Tested GAs were reported in the gut, haemolymph, and remaining tissues together (mainly the fat body and Malpighian tubules), with the highest percentage in the latter. The present study raises the possibility that SOL and CHA are not hydrolysed in the larvae of *T. molitor* by glycosidases because none of the hydrolysis products were detected in the tested samples. One possible mechanism for the detoxification of GAs is oxidation and/or sequestration. On the other hand, the GA concentration was the highest in the haemolymph. The SOL and CHA concentrations decreased in the haemolymph during the experiment, while they did not change in other tissues. Thus, they may be excreted by Malpighian tubules, with faeces or with cuticles during moulting. Moreover, GA excretion processes are relatively slow because 24 h is not enough to remove all the applied GAs from the larvae. Despite this, there were no lethal effects during the 10 days following GA administration. The rate of CHA elimination in the entire insect was the highest immediately after injection (0–0.5 h), while SOL was eliminated the fastest later (between 0.5–1.5 h). The presented results are significant because they facilitate the interpretation of the conducted research and future research related to the effects of GAs on insect metabolism. Further work is needed to explore the longer-term excretion of GAs in insects, as well as to evaluate the impact of the way in which insects are exposed to GAs on the detoxification of these compounds.

## Methods

### Insects

Larvae of *T. molitor* were obtained from colonies cultured at the Department of Animal Physiology and Developmental Biology at the Faculty of Biology of Adam Mickiewicz University in Poznań, Poland, at constant temperature (26 ± 0.5 °C), humidity (65 ± 5%), and a photoperiod of 12:12 h light to dark. The food consisted of oat flakes and fresh carrots. Only 15th- to 16th-instar larvae weighing approximately 120 to 140 mg were selected for the experiments.

### Compounds and treatment procedure

Saline solutions of synthetic SOL (≥ 95.0%, Cat. No. S3757) and CHA (≥ 95.0%, Cat. No. PHL80075) (Sigma‒Aldrich, Merck, Darmstadt, Germany) were used in experiments at a concentration of 10^−5^ M. The insects were injected with 2 μL of GA solution, which corresponds to 69.45 ng of SOL or 68.17 ng of CHA per sample composed of 4 larvae (with a dosage ranging from 0.12–0.14 ng/mg body mass). This concentration was selected based on the literature, the content of the plant extract, and our previous studies showing that it leads to different metabolic and developmental disorders^15,16,18,19^. The tested compounds were administered to larvae by injection using a microsyringe (Hamilton). The injection was made on the abdominal side of the larvae behind the last pair of legs after 8 min of CO_2_ anaesthesia.

### Tissue isolation and sample preparation for MS analyses

Samples of selected tissues (haemolymph (H), gut (G), and the rest of the larval body (FB), which mainly consists of the fat body) were isolated 0.5, 1.5, 8, and 24 h after GA injection. Before isolation, the larvae were anaesthetised with CO_2_. Our preliminary studies showed that the greatest changes in GA content were observed 24 h after GA injection. We chose these tissues because of their role in the distribution, metabolism, and detoxification of xenobiotics within the insect body^25,42^. Haemolymph was collected using an automatic pipette after the legs of the first pair were cut. After decapitation and removal of the last segment of the abdomen, the gut was isolated. The guts were not cleaned of food residuals. The rest of the larval body was then placed in Eppendorf tubes. The isolation was performed on ice to avoid sample degradation. After isolation, the gut and fat body samples were weighed to determine the fresh tissue mass, and the volume and weight of the haemolymph in each sample were measured. Next, the samples were homogenised in freshly prepared extraction buffer (methanol, 1% acetic acid with 1 µg/mL daidzein) using a pestle homogeniser (Fisherbrand, Ottawa, ON, Canada) and mixed at RT OV with a laboratory cradle (KL-942). Finally, the samples were centrifuged (10,000 RPM, 20 min, 4 °C) and filtered through syringe filters (0.22 µm), after which the supernatant was transferred to a new tube for LC-HRMS analysis.

### LC-HRMS analyses

Samples of isolated tissue extracts were transferred (0.5 ml) into vials for LC/MS analysis (Mini-UniPrep® syringeless filters with 0.2 µm pore size, PTFE membrane, Whatman), analysed with an LC system equipped with a photodiode array detector (Dionex) and coupled to a Q-exactive mass spectrometer (Thermo Fisher Scientific). LC separation was performed with (A) water (0.1% formic acid) and (B) 90:10 acetonitrile:H_2_O (0.1% formic acid) by injecting 5 µL of sample on a C18 Luna column (Phenomenex) with a 2.1 × 100 mm 2.5 μm particle size. The column oven temperature was set at 40 °C. The total run time was 32 min, and the flow rate was 0.250 ml/min. The elution system was as follows: 0 to 0.5 min 95% A/5% B, 24 min 25% A/75% B, and 26 min 95% A/5%, as previously described^43^. Ionisation was achieved by heating electrospray ionisation (HESI) in both positive and negative ionisation modes. The sheath and auxiliary gas were 40 and 10 units, respectively. The probe heater temperature was 330 °C, the capillary temperature was 250 °C, and the S-lens RF level was set at 50. The acquisition was performed in the mass range 110–1600 m/z both in positive and negative ion modes with the following parameters: resolution 70,000, microscan 1, AGC target 1 × 10^6^, and maximum injection time 50. SOL and CHA were quantified by LC–MS in HESI positive ionisation mode, integrating the area of the M + H ions of m/z 868.5053 and 852.5104 m/z (Dppm < 3), respectively, using calibration curves established with the analytical standards SOL (≥ 95.0%, Cat. No. S3757) and CHA (≥ 95.0%, Cat. No. PHL80075) and normalising the weight of the tissue used for the extraction. Standard solutions were prepared in methanol with 1% acetic acid at a concentration of 50 ng/ml and then serially diluted to working concentrations. All the solvents used were LC‒MS grade (Merck, Darmstadt, Germany).

### Survivability

The survival of *T. molitor* larvae during 10 days after GA injection was determined. The numbers of living and dead larvae were recorded every day for each experimental variant and each repetition. Each experiment was repeated three times with 15 larvae per replicate.

### Statistical analysis

Statistical calculations were made using GraphPad Prism 8.0.1, two-way ANOVA, and the log-rank test (Mantel‒Cox). The normality of the data was checked with the Shapiro‒Wilk test.

## Data Availability

The data analysed during this study are included in this published article.

## Abbreviations

GAs: Glycoalkaloids
SOL: α-Solanine
CHA: α-Chaconine
G: Gut sample
H: Haemolymph sample
FB: Sample prepared with whole larvae without gut and haemolymph
